# Announcing the Winner of the 2018 Maimonides Best Published Original Research Prize

**Published:** 2019-01-28

**Authors:** Shraga Blazer

**Affiliations:** Editor-in-Chief, Rambam Health Care Campus, Haifa, Israel

I am pleased to announce that the winner of the 2018 Maimonides Best Published Original Research Prize is Dr. Louise Kezerle, the first author of the paper entitled, “A Population-based Study of Peripartum Cardiomyopathy in Southern Israel: Are Bedouin Women a New High-risk Group?“ with co-authors Iftach M. Sagy, Leah Shalev, Offer Erez, and Leonid Barski.

The award ceremony will be held in due course, at Rambam Health Care Campus, Haifa, Israel. An announcement will be made once the date and venue have been set. The award ceremony will be open to the public.

This paper uncovered an increased risk for peripartum cardiomyopathy in Bedouin women. The knowledge that Bedouin women are a high-risk group for this well-known complication of pregnancy has the potential to save many lives.

We would like to congratulate the authors for their outstanding research that will have a positive impact on this Israeli community, and inspire original research that impacts diverse, albeit small demographic groups.

Determining a winner for this year’s prize was quite difficult. The four judges had to review 12 original research papers published in the past year—all of which were more than excellent. Obviously, only one paper could be selected for the prize; however the judges wished, in this announcement, to express their appreciation for the high quality of all of the submissions.

The Editorial Board is proud of the accomplishments of this small journal since its launch in July, 2010. We encourage our readership to submit their own papers, particularly review articles and original research, to *Rambam Maimonides Medical Journal*, for the benefit of the worldwide scientific medical community, and look forward to another winner in 2019.

Shraga Blazer, MD

Editor-in-Chief, *Rambam Maimonides Medical Journal*

For the Editorial Board

